# Dried Ginger Milk Extract Alleviates Inflammatory Bowel Disease-Associated Bone Loss via Gut Microbiota–Metabolite Remodeling and MEK/ERK Inhibition

**DOI:** 10.3390/ph19050675

**Published:** 2026-04-26

**Authors:** Yalan Li, Xuyang Liao, Chen Wang, Xingyu Bao, Yan Liu, Sufang Duan, Jian He, Jun Xu, Juan Wu, Mengyu Zhou, Guiying Peng

**Affiliations:** 1 School of Life Sciences, Beijing University of Chinese Medicine, Beijing 102488, China; liyalan@bucm.edu.cn (Y.L.); xuyangliao@bucm.edu.cn (X.L.); 20240941110@bucm.edu.cn (C.W.); 2 National Dairy Technology Innovation Center, Hohhot 010100, China; baoxingyu@yili.com (X.B.); liuyan37@yili.com (Y.L.); duansufang@yili.com (S.D.); hejian@yili.com (J.H.); xujun01@yili.com (J.X.); wujuan4@yili.com (J.W.); 3 Inner Mongolia Yili Industrial Group Co., Ltd., Hohhot 010100, China; 4 Department of Dentistry, The First Affiliated Hospital of Guangxi Medical University, Nanning 530021, China; zhoumengyu@gxmu.edu.cn

**Keywords:** dried ginger milk extract, inflammatory bowel disease, bone loss, gut microbiota, Th17 cells, MEK/ERK signaling

## Abstract

**Background:** Inflammatory bowel disease (IBD) is frequently complicated by secondary bone loss driven by chronic inflammation and gut–bone axis dysregulation. Although dried ginger has pharmacological activities relevant to intestinal inflammation, the effects of dried ginger milk extract (DGME), a lipophilic constituent-enriched preparation, on IBD-associated bone loss (IBD-BL) remain unknown. This study evaluated the preventive and therapeutic effects of DGME on IBD-BL and explored the underlying mechanisms. **Methods:** Mice with DSS-induced IBD-BL were treated with DGME (250, 125, or 62.5 mg/kg) or sulfasalazine. Colitis severity, bone microarchitecture, osteoclast activity and Th17 cells were assessed by histology, micro-computed tomography, histomorphometry and flow cytometric analysis. UHPLC-Q-TOF MS, network pharmacology, 16S rRNA sequencing, fecal metabolomics, and in vitro assays were used for mechanistic investigation. **Results:** DGME ameliorated colitis, improved trabecular bone microarchitecture, and reduced osteoclast-related bone destruction. These effects were associated with selective suppression of pathogenic bone marrow TNF-α^+^ Th17 cells and downregulation of *Il17a*, *Rorc*, *Tnfα*, *Ccr2*, *Ccr6*, *Cxcr4*, *Csf1*, and *Tnfsf11*. Compared with aqueous extract, DGME was enriched in 19 lipophilic constituents. Multi-omics analyses showed that DGME remodeled gut microbiota and metabolite profiles, characterized by enrichment of *Lactobacillus*, *Anaerotruncus*, vanillin, and spermidine. Both vanillin and spermidine suppressed Th17 effector genes and inhibited MEK/ERK signaling in vitro. **Conclusions:** DGME alleviated IBD-BL by suppressing pathogenic TNF-α^+^ Th17 responses and remodeling the gut microbiota–metabolite axis. This study not only extends the therapeutic application of dried ginger from intestinal inflammation to IBD-BL, but also identifies vanillin and spermidine as candidate functional mediators linked to MEK/ERK inhibition.

## 1. Introduction

Inflammatory bowel disease (IBD), comprising ulcerative colitis (UC) and Crohn’s disease (CD), is a chronic relapsing inflammatory disorder with substantial systemic consequences [1]. IBD-associated bone loss (IBD-BL) is a common extraintestinal complication characterized by reduced bone mineral density, compromised microarchitecture, and increased fracture risk [2]. Epidemiological evidence indicates that 18–42% of IBD patients develop osteopenia or osteoporosis, with significantly elevated fracture rates [3,4,5]. Currently, anti-inflammatory therapies, particularly glucocorticoids, can control intestinal inflammation but may simultaneously aggravate bone metabolic disorders [6,7]. Therefore, therapeutic strategies capable of attenuating intestinal inflammation while preserving skeletal homeostasis are urgently needed.

Dried ginger (*Zingiber officinale* Roscoe) is a traditional medicinal and edible herb with potential value for treating coupled intestinal inflammation and bone deterioration. Its lipophilic constituents, including gingerols and shogaols, exert multitarget effects on epithelial barrier function, immune regulation, and bone remodeling [8,9]. These compounds increase tight junction protein expression to strengthen intestinal barrier integrity. They reshape gut microbial composition and suppress colonic inflammation by inhibiting NLRP3 inflammasome activation and Th17 polarization [8,9]. Additionally, ginger-derived phytochemicals inhibit RANKL-mediated osteoclast differentiation through NF-κB suppression, while enhancing osteogenic activity via Wnt/β-catenin activation [10,11]. Such dual actions on intestinal and skeletal pathology render dried ginger a promising candidate for IBD-BL treatment.

The pathogenesis of IBD-BL involves interconnected immunological, microecological, and metabolic disturbances along the gut–bone axis. TNF-α^+^ T cells, particularly pathogenic TNF-α^+^ Th17 cells, can migrate from the inflamed intestine to bone and actively contribute to inflammatory bone loss [12,13]. At the skeletal level, the MAPK pathway, especially the RANKL downstream MEK/ERK axis, promotes osteoclast differentiation by driving c-Fos- and NFATc1-dependent transcriptional programs [14,15]. Concurrently, microbial-derived metabolites are important regulators of systemic immune homeostasis and osteoclast function [16]. Germ-free conditions, antibiotic-induced dysbiosis, and fecal microbiota transplantion have all been shown to alter bone mass and osteoclast dynamics [17,18]. In IBD, depletion of beneficial short-chain fatty acid (SCFA) bacteria (e.g., *Faecalibacterium*, *Roseburia*) impairs bone homeostasis, whereas expansion of pathobionts (e.g., *Escherichia*, *Shigella*) aggravates systemic inflammation and bone resorption [19,20]. Conversely, *Lactobacillus* supplementation suppresses osteoclastogenesis [21]. These microbial alterations are accompanied by metabolic disturbances, including reduced SCFAs, indoles, and polyamines, alongside increased purine intermediates and oxidative stress-related metabolites [22,23,24]. However, how microbiota-derived metabolite remodeling modulates TNF-α^+^ Th17 responses and contributes to alleviating IBD-BL remains unclear.

The extraction strategy is a critical determinant of the pharmacological activity of ginger preparations. Conventional aqueous extraction primarily recovers hydrophilic components, whereas key lipophilic constituents (e.g., shogaols) exhibit limited extraction efficiency and poor bioavailability. By contrast, milk-based extraction improves the solubility, stability, and intestinal delivery of lipophilic phytochemicals through protein–lipid interactions, potentially enhancing their in vivo efficacy [25,26]. Whether dried ginger milk extract (DGME) provides therapeutic advantages over conventional aqueous extract in IBD-BL remains uninvestigated. More importantly, how DGME modulates immune responses and gut microbial metabolism remains undefined.

Therefore, this study evaluated the therapeutic efficacy of DGME in DSS-induced IBD-BL and elucidated how DGME reshapes gut microbiota and metabolites to suppress TNF-α^+^ Th17 cells and inhibit MEK/ERK signaling.

## 2. Results

### 2.1. DGME Alleviates Colitis and Prevents Bone Loss in DSS-Induced IBD-BL Mice

**Figure 1:**
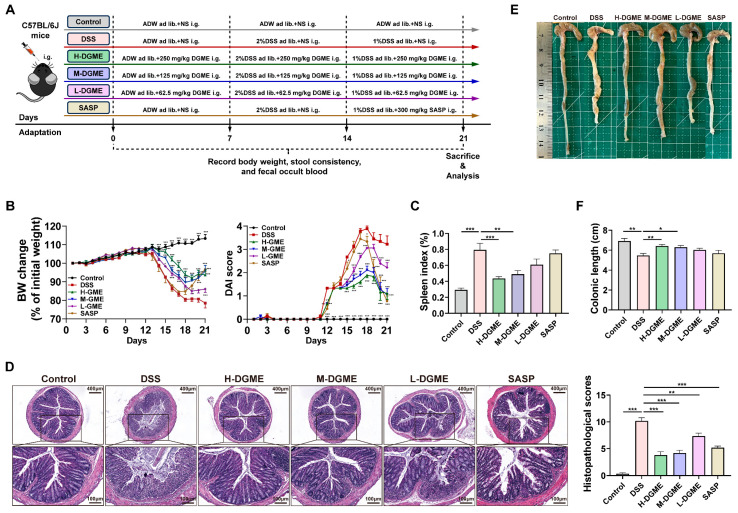
DGME alleviates DSS-induced colitis of IBD-BL mice. (**A**) The time course of DSS administration and different treatments in mice. (**B**) Body weight (BW) changes in mice throughout the entire trial and DAI scores in each group. (**C**) Spleen index. (**D**) H&E staining of the colon and quantitative analysis of colonic histopathology. (**E**) Representative images of colons. (**F**) Colonic length in each group. Data are presented as mean ± SEM. Statistical significance was determined by comparison with the DSS group. * *p* < 0.05, ** *p* < 0.01, *** *p* < 0.001.

We first established an IBD-BL model as shown in Figure 1A to evaluate the preventive and curative effects of DGME. DSS challenge induced progressive body weight loss, elevated DAI score, and increased spleen index (Figure 1B,C). H&E staining of colonic sections revealed extensive mucosal damage in the DSS group. This damage was characterized by epithelial disruption, crypt architectural distortion, goblet cell depletion, and dense inflammatory infiltration in mucosal and submucosal layers. Consequently, DSS-treated mice exhibited significantly increased histopathological scores and marked colon shortening compared to controls (Figure 1D–F). These alterations were ameliorated by DGME treatment, particularly at the high and medium doses, as well as by SASP administration (Figure 1B–F).

**Figure 2:**
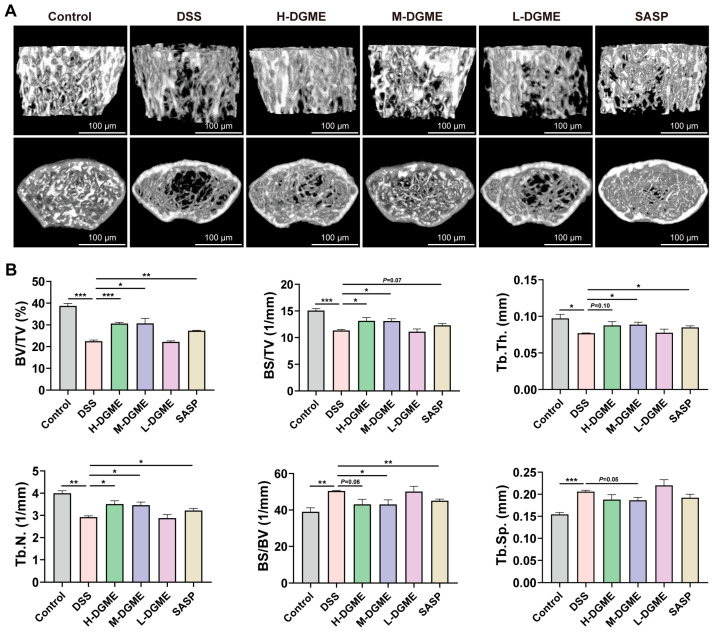
DGME prevents bone loss in DSS-induced IBD-BL mice. (**A**) Representative micro-CT three-dimensional reconstruction of distal femoral trabecular bone. (**B**) Quantitative micro-CT analysis of trabecular bone parameters, including bone volume fraction (BV/TV), bone surface density (BS/TV), trabecular thickness (Tb.Th.), trabecular number (Tb.N.), bone volume ratio (BS/BV), and trabecular separation (Tb.Sp.). Data are presented as mean ± SEM. Statistical significance was determined by comparison with the DSS group. * *p* < 0.05, ** *p* < 0.01, *** *p* < 0.001.

Concomitant with intestinal inflammation, DSS-treated mice developed marked trabecular bone deterioration. Micro-CT analysis revealed compromised bone microarchitecture manifested by reduced BV/TV, BS/TV, Tb.N., and Tb.Th., together with increased BS/BV and Tb.Sp., indicating prominent loss of trabecular bone mass and connectivity (Figure 2A,B). Both H-DGME and M-DGME significantly enhanced BV/TV, BS/TV, and Tb.N. (Figure 2A,B). M-DGME additionally improved Tb.Th., Tb.Sp., and BS/BV. Thus, the medium dose exhibited more comprehensive bone protection and was selected in subsequent mechanistic studies.

### 2.2. DGME Suppresses Pathogenic Th17-Driven Inflammatory Bone Loss in IBD-BL Mice

**Figure 3:**
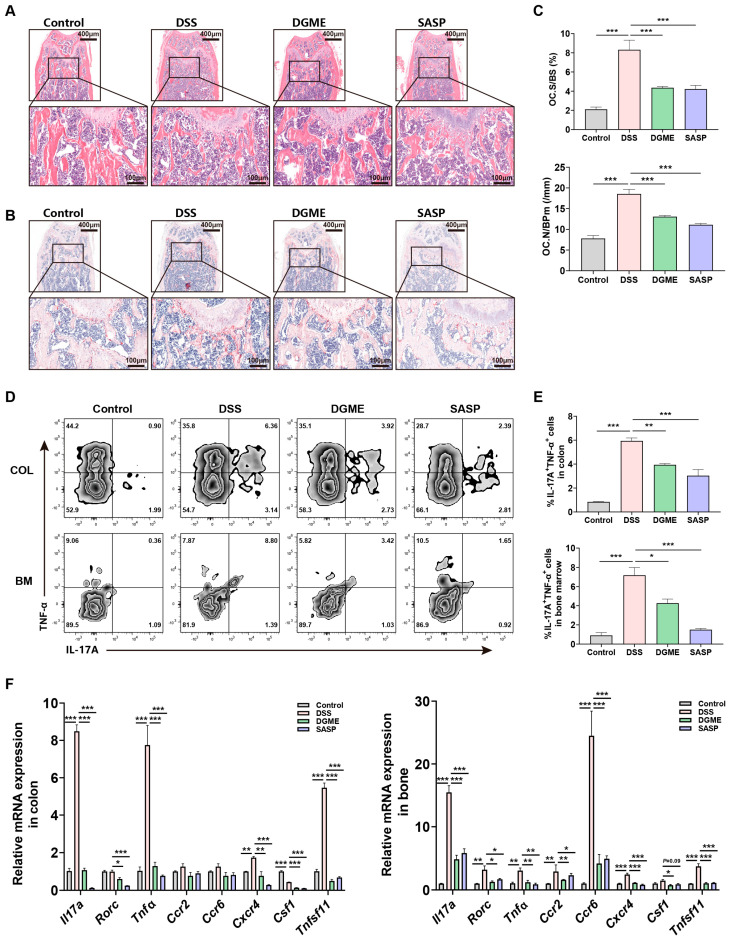
DGME attenuates pathogenic Th17-mediated inflammatory bone pathology in IBD-BL mice. (**A**) Representative H&E staining of distal femoral sections showing bone marrow architecture and trabecular structure. (**B**,**C**) Representative TRAP staining of distal femur sections. TRAP-positive multinucleated cells (≥3 nuclei) with wine-red cytoplasm were counted as mature osteoclasts (**B**) OC. N/BPm (osteoclast number per bone parameter) and OC. S/BS (osteoclast surface per bone surface) were determined (**C**). (**D**,**E**) Representative scatter plots for identifying TNF-α^+^ Th17 cells (CD3^+^ CD4^+^ IL-17A^+^ TNF-α^+^) in colon (COL) and bone marrow (BM) quantified by flow cytometry (**D**). The proportions of TNF-α^+^ Th17 cells (**E**). (**F**) Relative mRNA expression of Th17-related and osteoclast-associated genes including *Il17a*, *Rorc*, *Tnfα*, *Ccr2*, *Ccr6*, *Cxcr4*, *Csf1* (M-CSF), and *Tnfsf11* (RANKL) in colon and bone tissue were assessed by RT-qPCR. Data are presented as mean ± SEM. Statistical significance was determined by comparison with the DSS group. * *p* < 0.05, ** *p* < 0.01, *** *p* < 0.001.

Histological evaluation of distal femora revealed severe trabecular bone loss in mice with IBD-BL. The marrow microenvironment also showed profound deterioration, including disrupted architecture, expanded cavities, and diminished hematopoietic tissue (Figure 3A). Consistently, TRAP staining demonstrated abundant multinucleated osteoclasts along trabecular surfaces in DSS mice. Quantitative analysis revealed significant increases in OC.N/BPm and OC.S/BS (Figure 3B,C). DGME treatment substantially ameliorated these alterations. It restored trabecular integrity and reduced both osteoclast number and surface area. The efficacy was comparable to SASP. These improvements indicate potent suppression of osteoclast activation and inflammatory bone resorption (Figure 3A–C).

Th17 cells, particularly pathogenic TNF-α^+^ Th17 cells, play a pivotal role in colitis and bone destruction [12,13]. These cells represent the core pathogenic immune population linking intestinal inflammation to bone loss. Following DSS challenge, we observed marked expansion of Th17 cells and pathogenic TNF-α^+^ Th17 cells in both the colon and bone marrow. DGME significantly diminished these populations in the colon. However, while DGME showed a trend toward reducing total Th17 cells, it significantly decreased TNF-α^+^ Th17 cells. These findings suggest that bone marrow pathogenic TNF-α^+^ Th17 cells may be more sensitive to DGME under the present experimental conditions (Figure 3D,E and Figure S1).

We further evaluated the effects of DGME on effector molecules mediating inflammatory, migratory, and osteoclastogenic functions. RT-qPCR analysis showed significant elevations of *Il17a*, *Tnfα*, *Cxcr4*, and *Tnfsf11* in colonic tissues. Pronounced induction of *Il17a*, *Rorc*, *Tnfα*, *Ccr2*, *Ccr6*, *Cxcr4*, and *Tnfsf11* was also observed in bone tissues (Figure 3F). DGME substantially attenuated these inflammatory transcripts in both compartments, suggesting broad suppression of Th17-associated osteoclastogenic signaling.

### 2.3. DGME Enriches Lipophilic Bioactive Constituents Relative to Aqueous Extraction

Accumulating evidence indicates that lipophilic constituents (such as gingerols and shogaols) in dried ginger confer protective effects against experimental colitis and osteoclastogenesis [8,9]. We conducted comparative metabolomic analysis based on UHPLC-Q-TOF MS to delineate chemical differences between DGME and dried ginger water extract (DGWE).

**Figure 4:**
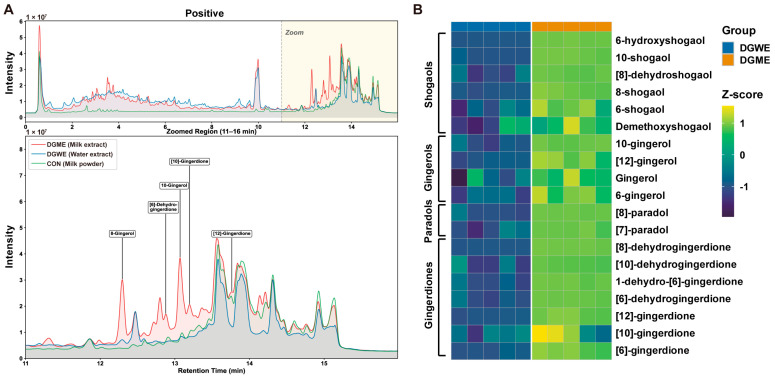
UHPLC-Q-TOF MS-based comparative metabolomic profiling of DGME and DGWE. (**A**) Total ion chromatograms of the three samples in positive ion mode. (**B**) Differentially abundant metabolites between DGME and DGWE obtained by LC-MS; compounds in heatmaps are colored based on a Z-score scale of metabolite abundances.

Positive ion mode analysis revealed broadly comparable total ion chromatogram profiles across the 0–16 min retention range. However, detailed examination of the late-eluting region (11–16 min) demonstrated markedly higher peak intensities for lipophilic ginger constituents in DGME. These included gingerols and gingerdione derivatives (e.g., 8-gingerol, 10-gingerol, 6-dehydrogingerdione, 10- and 12-gingerdione). Peak increases ranged approximately 2–5-fold relative to DGWE (Figure 4A). Conversely, negative ion mode analysis revealed higher abundances of early-eluting polar phenolic acids and flavonoids in DGWE within the 2–8 min region. Representative constituents included 3,4-dihydroxyphenylacetic acid, luteolin 4′-glucoside, 1,3-dicaffeoylquinic acid, acetyleugenol, and coniferyl ferulate (Figure S2).

Notably, DGME retained detectable levels of these hydrophilic metabolites while significantly enriching lipophilic bioactives. This pattern was corroborated by heatmap visualization of 19 lipophilic constituents (Figure 4B, Table 1). Although previous studies have reported that DGWE can alleviate ulcerative colitis [27], our Supplementary Materials showed that only DGME produced a clearer protective effect on IBD-BL-related bone parameters under the present experimental conditions, whereas DGWE showed only a trend toward improvement (Figure S3). This differential efficacy may be attributed to substantial enrichment of lipophilic bioactive compounds in the milk extraction process [28]. These results indicate that the milk-based extraction preferentially enriches lipophilic ginger constituents without fully depleting phenolic acids and flavonoids. Such chemical composition provides a robust foundation for the observed pharmacological effects.

### 2.4. Network-Based Identification of MEK/ERK Signaling as a Key Pathway of DGME

**Figure 5:**
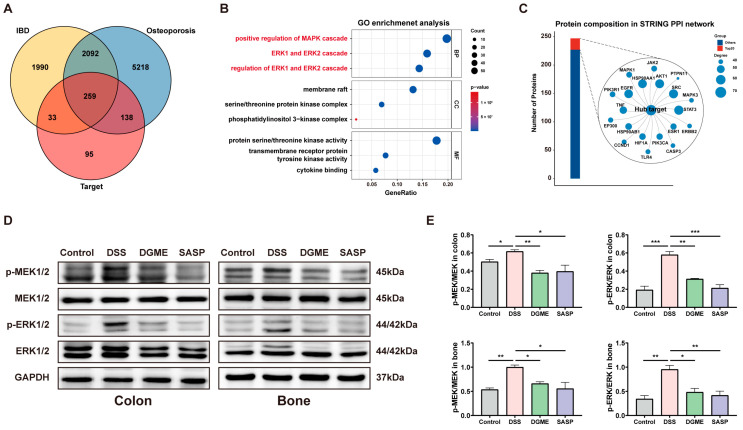
Network-based identification and experimental validation of MEK/ERK signaling as a key pathway of DGME. (**A**) Venn diagram showing the intersection of DGME-, IBD-, and osteoporosis-related targets. (**B**) GO enrichment analysis. (**C**) PPI network and hub target identification. (**D**,**E**) Western blot analysis and quantification of MEK1/2 and ERK1/2 phosphorylation in colon and bone tissues (p-MEK/MEK and p-ERK/ERK). Data are presented as mean ± SEM. Statistical significance was determined by comparison with the DSS group. * *p* < 0.05, ** *p* < 0.01, *** *p* < 0.001.

A total of 19 lipophilic constituents were identified in DGME through differential phytochemical analysis. Following structural verification via PubChem, SwissTargetPrediction yielded 525 putative targets, enabling construction of a compound–target interaction network (Figure S4A). Jaccard similarity analysis indicated minimal target overlap among individual constituents. This supports a complementary multi-component, multi-target pharmacological paradigm (Figure S4B). GeneCards and DiseaseNet queries retrieved 4374 disease-associated targets for IBD and 7707 for osteoporosis, respectively. Intersection analysis identified 259 shared targets constituting the core drug–disease interaction network (Figure 5A). Functional enrichment analysis revealed significant pathway convergence. KEGG analysis highlighted prominent involvement of PI3K-AKT, MAPK signaling pathways, and osteoclast differentiation (Figure S4C). Concurrently, GO enrichment emphasized ERK1/ERK2-centered MAPK regulation and kinase-associated biological processes (Figure 5B). STRING-based PPI network construction identified densely interconnected hub nodes. These included JAK2, STAT3, MAPK1, MAPK3, AKT1, EGFR, SRC, and TNF (Figure 5C). Western blot analysis confirmed aberrant MEK1/2 and ERK1/2 phosphorylation in both colonic and bone tissues following DSS challenge (Figure 5D,E). DGME administration markedly suppressed these phosphorylation events. It significantly reduced p-MEK/MEK and p-ERK/ERK ratios in the colon and achieved comparable suppression in bone tissue. The efficacy was similar to SASP. These findings identify MEK/ERK as the core pharmacological axis of DGME. They validate its multi-target mechanism against IBD-BL.

### 2.5. DGME Selectively Remodels DSS-Induced Gut Microbial Dysbiosis

**Figure 6:**
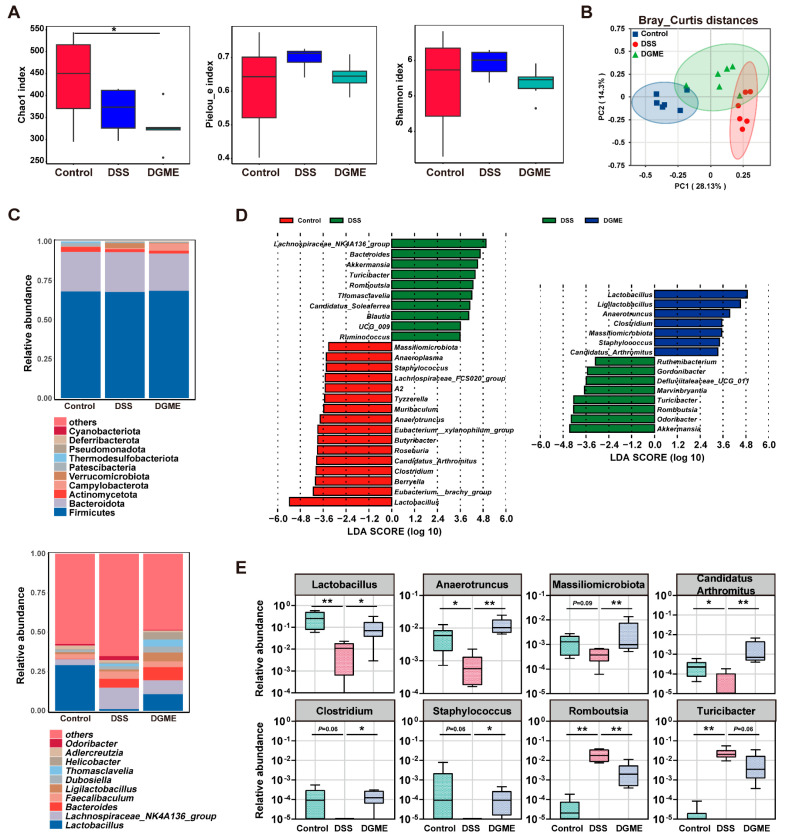
Overall gut microbiota structure and differentially abundant microbes in the feces among different groups. (**A**) Alpha-diversities of gut microbiota profiles calculated using the Chao1, Pielou_e and Shannon indices. (**B**) Beta-diversities of gut microbiota profiles illustrated with PCoA using Bray–Curtis distances. (**C**) Abundant phyla and genera. (**D**) Differentially abundant genera identified by LEfSe with a linear discriminant analysis (LDA) score (threshold > 2). (**E**) Relative abundance of differential genera in each group. Data are presented as mean ± SEM. Statistical significance was determined by comparison with the DSS group. * *p* < 0.05, ** *p* < 0.01.

We further examined the effects of DGME intervention on gut microbiota in IBD-BL mice. At the overall community level, IBD-BL mice with or without DGME intervention exhibited smaller Chao1, Pielou_e and Shannon indices compared to controls (Figure 6A). This indicates a lower community richness and evenness. Principal coordinate analysis (PCoA) plots coupled with PERMANOVA further revealed significant separation of fecal samples among the three groups based on Bray–Curtis (R^2^ = 0.361, *p* = 0.001, Figure 6B) and Jaccard distances (R^2^ = 0.315, *p* = 0.001, Figure S5A). Significant differences in community composition were also observed among groups (Figure 6C).

Subsequently, we conducted LEfSe analysis (LDA > 2) to identify differentially abundant genera. As shown in Figure 6D,E, several genera depleted in IBD-BL mice compared to controls were significantly restored following DGME administration. These included *Lactobacillus*, *Anaerotruncus*, *Massiliomicrobiota*, *Candidatus_Arthromitus*, *Clostridium*, and *Staphylococcus*. Conversely, *Romboutsia* and *Turicibacter* exhibited elevated relative abundance in the DSS group compared to controls. DGME markedly suppressed these pro-inflammatory taxa. These findings demonstrate that DGME ameliorates DSS-induced dysbiosis through selective modulation of key commensal and pro-inflammatory genera. This underscores the precision therapeutic potential of DGME in gut microbiota remodeling.

### 2.6. Metabolic Remodeling by DGME in IBD-BL Mice

**Figure 7:**
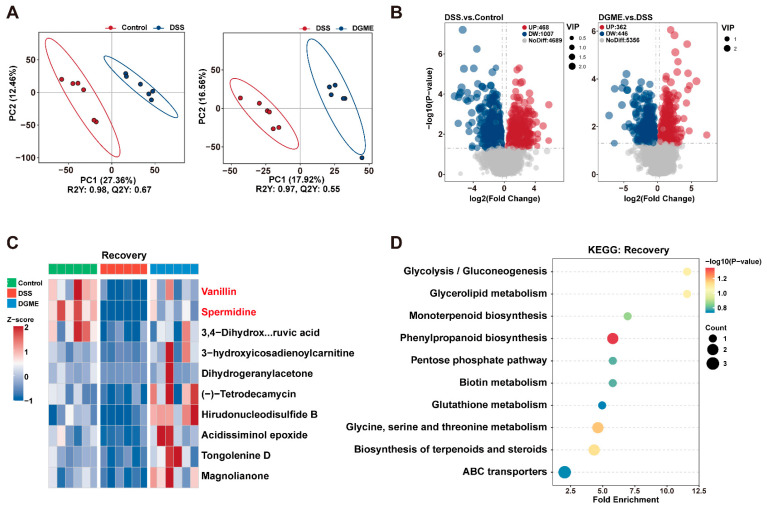
DGME remodels fecal metabolic profiles of IBD-BL mice. (**A**) PLS-DA plots of fecal metabolites obtained by untargeted metabolomics. (**B**) Volcano plots of differential metabolites. (**C**) Z-score heatmap of recovery-pattern metabolites. (**D**) KEGG enrichment analysis of recovery metabolites. Differential metabolites were screened using *p* < 0.05, VIP > 1.0 and fold change > 1.2 or <0.833.

To assess the microbial metabolic impact of DGME on IBD-BL mice, we undertook untargeted fecal metabolomics to roughly profile the potential metabolite among the three groups. Partial least squares discriminant analysis (PLS-DA) revealed significant segregation between every two groups of participants (Figure 7A). Differential metabolite analysis further revealed widespread metabolic alterations. DSS treatment induced significant perturbations compared with controls. Specifically, 468 metabolites were upregulated and 1007 were downregulated. Conversely, DGME reversed these alterations. It upregulated 362 metabolites and downregulated 446 metabolites relative to the DSS group, indicating partial restoration of metabolic homeostasis (Figure 7B). These findings collectively demonstrate that DGME effectively ameliorates DSS-induced metabolic dysregulation.

Next, we performed therapeutic pattern screening by integrating untargeted metabolomics profiles across the three groups. Metabolites were classified into Pattern A (Recovery) and Pattern B (Suppression). Pattern A comprised metabolites suppressed during DSS-induced colitis and restored by DGME. Pattern B comprised metabolites elevated during colitis and normalized following treatment. We identified 145 recovery-pattern and 114 suppression-pattern metabolites. The top 10 compounds for each pattern are shown in Figure 7C and Figure S5B. DSS reduced microbiota-associated metabolites such as vanillin (VAN) and spermidine (SPD), while increasing GMP and cGMP. These metabolic alterations were largely reversed following DGME intervention.

KEGG enrichment analysis revealed distinct pathway associations. Pattern A metabolites were primarily associated with phenylpropanoid biosynthesis and plant secondary metabolite pathways (Figure 7D). In contrast, Pattern B metabolites were significantly enriched in the cGMP-PKG signaling, purine metabolism, and nucleotide metabolism (Figure S5C).

Parallel target prediction for gingerol-type constituents was performed using the TCMSP database. This analysis yielded 164 significantly enriched pathways. These included IL-17, TNF, NF-κB, MAPK, and PI3K-AKT signaling. Notably, cGMP-PKG signaling was also enriched among drug targets (Figure S5D). Cross-referencing revealed cGMP-PKG as the top convergent pathway between drug targets and fecal metabolites. This suggests a mechanistic link between DGME constituents and colonic metabolic alterations.

### 2.7. Vanillin and Spermidine Mediate DGME Suppression of MEK/ERK Signaling

**Figure 8:**
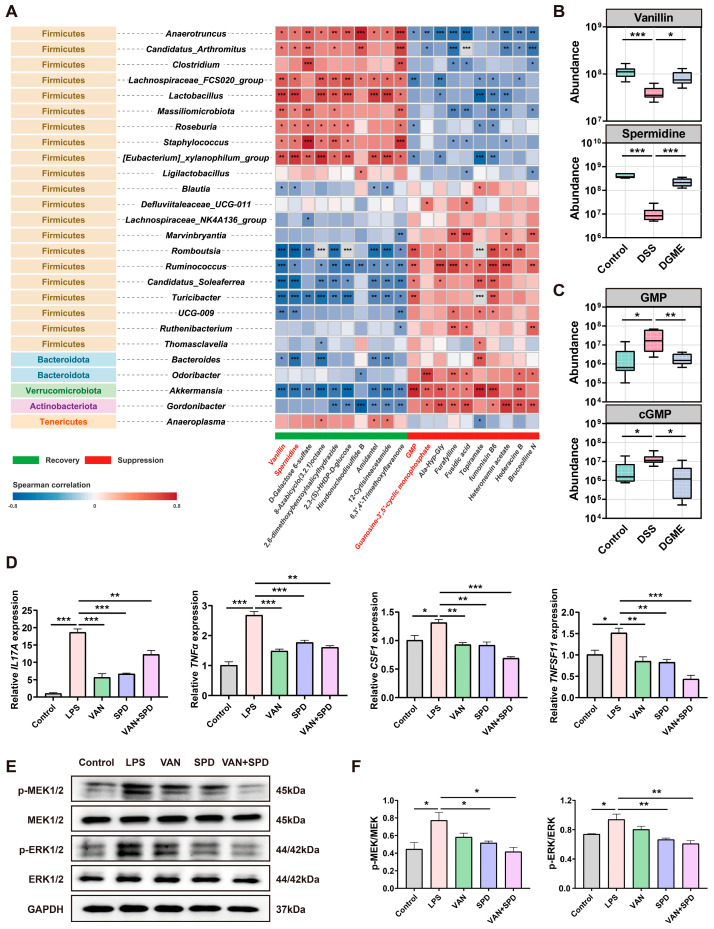
DGME promotes microbiota-associated VAN and SPD to inhibit MEK/ERK signaling. (**A**) Spearman correlation analysis between differential genera and metabolites. (**B**,**C**) Targeted quantification of VAN, SPD, GMP, and cGMP. (**D**) Relative mRNA expression of *IL17A*, *TNFα*, *CSF1*, and *TNFSF11* in Jurkat T cells was assessed by RT-qPCR. (**E**,**F**) Western blot analysis and quantification of MEK and ERK phosphorylation. Data are presented as mean ± SEM. Statistical significance was determined by comparison with the LPS group. * *p* < 0.05, ** *p* < 0.01, *** *p* < 0.001.

Spearman correlation analysis demonstrated a coordinated interaction between the gut microbiota and metabolites. This interaction underlies DGME-induced metabolic reprogramming (Figure 8A). Recovery-pattern metabolites, including VAN and SPD, showed positive associations with commensal taxa. These included *Lactobacillus*, *Roseburia*, *Anaerotruncus*, *Candidatus_Arthromitus*, *Massiliomicrobiota*, and *Lachnospiraceae_FCS020_group*. Conversely, suppression-pattern metabolites (GMP and cGMP) correlated positively with DSS-enriched genera, including *Ruminococcus*, *Turicibacter*, and *Romboutsia*. Targeted quantification further validated these alterations (Figure 8B,C). DSS markedly reduced VAN and SPD levels while elevating GMP and cGMP. DGME intervention significantly normalized all these metabolites. These findings indicate that DGME reshapes gut metabolic output through selective modulation of key microbial taxa.

Microbial biotransformation of gingerols generates vanillic acid. This compound is structurally related to VAN and alleviates colitis through NF-κB suppression [29,30]. SPD is a pleiotropic polyamine with anti-aging and autophagy-promoting activities. It attenuates both intestinal inflammation and osteoclast differentiation via the same pathway [31,32]. CCK-8 cytotoxicity assay determined non-toxic doses for subsequent experiments (Figure S6A,B; Tables S3 and S4). We then investigated the anti-inflammatory effects of VAN and SPD in LPS-stimulated Jurkat T cells. Both metabolites significantly downregulated the expression of *IL17A*, *TNFα*, *CSF1*, and *TNFSF11* (Figure 8D). This indicates concomitant attenuation of inflammatory responses and osteoclastogenic factor production. Western blot analysis showed that SPD and the combination treatment (VAN + SPD) markedly suppressed MEK and ERK phosphorylation. VAN alone did not produce this effect (Figure 8E,F). This finding indicates specific inhibition of the MAPK cascade by SPD, or synergistic effects achieved with halved doses. Collectively, these data indicate that DGME-derived metabolites inhibit MEK/ERK signaling. This inhibition attenuates inflammatory and osteoclastogenic responses in IBD-BL.

## 3. Discussion

IBD-BL is increasingly recognized as a multifactorial extraintestinal complication involving chronic inflammation, immune dysregulation, metabolic disturbance, and gut microbiota imbalance [7]. Persistent intestinal inflammation elevates osteoclastogenic cytokines such as TNF-α, IL-6, and IL-17A. These cytokines enhance RANKL signaling and promote osteoclast differentiation. Consequently, bone mineral density decreases and fracture risk increases in IBD patients [33]. Concurrently, depletion of beneficial SCFA-producing genera and expansion of pro-inflammatory taxa aggravate systemic inflammation and osteoclastogenic signaling [16,34,35]. These microbial alterations also reshape key metabolites, including butyrate, indole derivatives, and polyamines. These metabolites regulate immune homeostasis and influence Th17/Treg balance. They further affect bone remodeling through NF-κB, JAK/STAT, and MAPK pathways [36,37,38]. Sustained MEK/ERK activation may amplify NFATc1-dependent osteoclastogenic programs [38].

Current therapies include TNF-α-targeting biologics and anti-resorptive agents such as bisphosphonates and RANKL inhibitors. These can partially slow bone deterioration in IBD. However, their efficacy remains limited and adverse effects are frequent [39,40]. Dried ginger has therefore attracted attention as a potential adjunctive therapy. Its major bioactive constituents exhibit anti-inflammatory, antioxidant, and immunomodulatory activities. They suppress NF-κB and MAPK signaling. They also modulate gut microbiota and restore intestinal barrier integrity. Additionally, they promote osteogenesis and inhibit RANKL-induced osteoclastogenesis [10,41,42,43,44]. Pathogenic TNF-α^+^ Th17 cells are closely linked to IBD severity and bone loss. These cells are driven by STAT3/RORγt-related inflammatory signaling, promote osteoclastogenesis and induce matrix-degrading factors. They also recruit inflammatory monocytes within the bone marrow niche [45,46,47,48,49,50,51]. The MEK/ERK pathway, as an important branch of MAPK signaling, supports Th17 pathogenic programming and effector function [52]. Consistent with this, our results showed that DGME preferentially reduced bone marrow TNF-α^+^ Th17 cells while largely preserving total Th17 cells. This effect was accompanied by marked inhibition of MEK/ERK signaling. Together, these findings support the possibility that MEK/ERK-associated TNF-α^+^ Th17 pathogenicity contributes to IBD-BL and may represent a therapeutic target of DGME.

Our central finding is that DGME reshapes the gut microbiota without altering overall diversity. Selective enrichment of beneficial commensals such as *Lactobacillus*, *Roseburia*, *Anaerotruncus* and *Lachnospiraceae_FCS020_group* may jointly inhibit pathogenic Th17 cell expansion. The *Lactobacillus* species (e.g. *Lactobacillus reuteri*) suppresses TNF-α production via cAMP/PKA-mediated MEK/ERK inhibition [53]. In contrast, *Roseburia*, *Anaerotruncus*, and *Lachnospiraceae_FCS020_group* are prolific producers of butyrate and valerate. These SCFAs inhibit HDACs to dampen Th17 differentiation while promoting Foxp3^+^ Treg induction, thereby restoring Th17/Treg balance [54]. Conversely, DGME-mediated reduction in pathogenic taxa alleviates Th17 polarization and TNF-α burden. These genera include *Candidatus_Arthromitus* (segmented filamentous bacteria), *Turicibacter*, and *Romboutsia*. *Candidatus_Arthromitus* drives Th17 activation via MHC-II-dependent antigen presentation and triggering serum amyloid A (SAA)-mediated IL-6/STAT3 signaling. *Turicibacter* and *Romboutsia* are associated with anxiety-like phenotypes and intestinal inflammation [55,56]. Notably, *Clostridium* exhibits context-dependent heterogeneity. Certain clusters promote Treg induction, whereas others contribute to dysbiosis-associated inflammation [57].

DGME-enriched beneficial bacteria showed positive correlations with VAN and SPD levels. In contrast, DSS-driven expansion of pathogenic taxa coincided with accumulation of nucleotide metabolites (GMP and cGMP). These metabolites function as critical executors mediating DGME’s immunomodulatory effects. They converge to suppress Th17-related cytokines (IL-17A, TNF-α) and osteoclastogenic factors (CSF1, TNFSF11) in activated T cells. SPD directly suppressed MEK/ERK phosphorylation. It also attenuated IL-17A and TNF-α production through autophagy induction and mitochondrial quality control. These actions counteract the sustained oxidative metabolism required for inflammatory persistence in pathogenic Th17 cells [58,59]. However, VAN, which is derived in part from intestinal microbial biotransformation of gingerols and has documented anti-colitic and antioxidant effects [30], did not directly inhibit the MEK/ERK signaling axis. This suggests that VAN operates through distinct, non-redundant pathways. Significant MEK/ERK suppression occurred only under combined treatment. This indicates that VAN potentiates SPD-mediated immunosuppression indirectly. VAN may achieve this by dampening upstream inflammatory signals (e.g., NF-κB activation) or by enhancing polyamine metabolism. Such actions create a cellular milieu permissive for SPD-driven pathway attenuation [60,61]. Alternatively, VAN may independently stabilize intestinal barrier integrity. It may also modulate pattern recognition receptor signaling to reduce the inflammatory load that drives MEK/ERK activation [62,63]. These findings indicate that DGME exerts therapeutic effects through the combinatorial metabolic interplay of VAN and SPD, which coordinately suppress pathogenic T-cell responses via complementary mechanisms. However, the precise molecular basis of this interaction remains to be further elucidated.

Notably, metabolomic profiling identified GMP and cGMP as major microbial metabolites enriched in the cGMP-PKG signaling pathway. Both were significantly reduced after DGME treatment. TCMSP-based target prediction and integrative pathway analysis further indicated that cGMP-PKG signaling was the top-ranked pathway. Reverse target mapping suggested that [10]-gingerdione, 10-gingerol, [12]-gingerol, and quercetin may modulate this pathway. These constituents potentially target ADRA1A, ADRA1B, ADRB1, ADRB2, AKT1, and MAPK1 [64]. These findings suggest that DGME-derived ginger constituents alleviate colonic inflammation through adrenergic receptor-associated cGMP-PKG signaling [65]. This mechanism links microbial metabolic reprogramming with pharmacological activity. Although we identified microbiota–metabolite correlations, the causal role of specific taxa in DGME-mediated bone protection remains to be validated. This requires fecal microbiota transplantation (FMT) in germ-free or gnotobiotic mouse models.

The milk-based extraction strategy likely plays a mechanistic role in amplifying these effects. Lipophilic gingerols, shogaols, paradols, and gingerdiones readily partition into milk-derived lipid matrices. These matrices act as natural nano-carriers, enhancing intestinal stability and microbial biotransformation [66]. These derivatives serve as substrates for microbial pathways generating vanillin and related metabolites that feed back into host immune regulation. This matrix-dependent modulation highlights that extraction vehicles actively reshape microbial metabolic trajectories. They fundamentally modulate systemic host responses through ecological metabolic effects rather than merely serving as bioactive carriers.

## 4. Materials and Methods

### 4.1. Preparation of DGME and DGWE

We selected DGME as the intervention agent based on its established therapeutic effects against ulcerative colitis in our previous study [28]. The DGME was manufactured and supplied by Inner Mongolia Yili Industrial Group Co., Ltd. (Hohhot, China) [28]. For oral administration, DGME was suspended in distilled water at 25, 12.5, and 6.25 mg/mL, and DGWE at 40 mg/mL. Mice received 200 μL per gavage. All suspensions were prepared freshly and administered immediately after preparation.

### 4.2. Animals and Treatment

Male C57BL/6 J mice (20–22 g), purchased from SPF Biotechnology Co., Ltd. (Beijing, China), were randomly divided into six groups (*n* = 6 per group): Control, DSS, DGME high-dose (H-DGME, 250 mg/kg), medium-dose (M-DGME, 125 mg/kg), low-dose (L-DGME, 62.5 mg/kg), and sulfasalazine (SASP, 300 mg/kg). For the comparison between DGME and DGWE, the latter was administered at 400 mg/kg according to our previous findings [67]. To induce IBD-BL, mice (except Control) received 2% (*w*/*v*) DSS (MP Biomedicals, MW: 36,000–50,000) ad libitum in drinking water for 7 days followed by 1% DSS for another 7 days (replaced every 2 days). Mice received DGME (250, 125, or 62.5 mg/kg) and DGME (400 mg/kg) by daily oral gavage from Day 0 to 21. DSS was administered in drinking water from Day 7 to 21. SASP was administered by gavage from Day 14 to 21. Mice in the Control and DSS groups were administered autoclaved drinking water (ADW). Each mouse was recorded daily for body weight loss, stool consistency, and fecal occult blood, and the DAI score was equal to the average of the above three indicators. The severity of colitis was evaluated using the DAI score as described previously [68]. At the end of the experiment, mice were sacrificed, and the colon and bone tissues were collected for further analysis. The timeline of this test is shown in Figure 1A. All procedures involving animals were approved by the Animal Ethics Committee of Beijing University of Chinese Medicine (Approval No. BUCM-2025041301-2024, Approval date: 24 April 2025), following guidelines issued by Regulations of Beijing Laboratory Animal Management.

### 4.3. Histopathological Evaluation

Colorectal tissues were fixed in 10% formalin for 48 h, paraffin-embedded, sectioned, and stained with hematoxylin and eosin (H&E). Tissue damage and inflammatory infiltration were scored as previously described [69]. Bilateral femurs were fixed in 10% neutral buffered formalin (72 h, 4 °C), decalcified in 10% EDTA-2Na (pH 7.0 ± 0.2, 4 °C) for 2–3 weeks with solution renewal every 2–3 days until complete decalcification. Specimens were dehydrated, cleared, and embedded in paraffin. Femoral sections (5 μm) were stained with H&E for examination of trabecular architecture, marrow cavity, and growth plates under light microscopy (200×).

### 4.4. Micro-Computed Tomography (Micro-CT) Analysis of Femurs

Femurs were collected after sacrifice, carefully cleared of soft tissue, and fixed in 4% paraformaldehyde for at least 48 h. Samples were scanned using a Bruker SkyScan 1276 micro-CT system (Bruker, Kontich, Belgium) under identical parameters: 55 kV, 200 µA, 0.25 mm Al filter, 120 ms exposure, 0.4° rotation step over 180°, frame averaging = 2, voxel size = 10.123 μm. Projection images were reconstructed with NRecon software (v2.2.0.6), and trabecular bone microarchitecture was analyzed using CTAn. The volume of interest (VOI) was defined in the distal femoral metaphysis beginning 200 slices above the growth plate and extending for 300 slices, with cortical bone manually excluded. Bone segmentation was performed using a global threshold (120–255). Morphometric parameters including bone volume fraction (BV/TV), bone surface-to-bone volume ratio (BS/BV), bone surface density (BS/TV), trabecular thickness (Tb.Th), trabecular number (Tb.N), structural model index (SMI) and trabecular separation (Tb.Sp) were calculated. Three-dimensional images were generated using CTVox for visualization.

### 4.5. Tartrate-Resistant Acid Phosphatase (TRAP) Staining

Femoral sections were stained for TRAP using a kit according to the manufacturer’s instructions (BaiQianDu Biotechnology, B1032, Wuhan, China) and incubated at 37 °C for 1–2 h. Following distilled water washes, sections were counterstained with hematoxylin, dehydrated through graded ethanol, cleared in xylene, and mounted with neutral resin. TRAP-positive multinucleated osteoclasts (wine-red cytoplasm, ≥3 nuclei) were identified under light microscopy. Four high-power fields (200×) in the femoral metaphysis were randomly selected for osteoclast (OC) counting. The number of OCs per field and the number of nuclei per TRAP-positive cells were quantified by ImageJ software (v1.54).

### 4.6. Real-Time Quantitative PCR (RT-qPCR)

Total RNA was isolated from frozen tissues (powdered in liquid nitrogen) or cells using TRIzol reagent, followed by chloroform extraction and isopropanol precipitation. After washing with 80% ethanol, RNA was dissolved in RNase-free water and quantified spectrophotometrically. Reverse transcription was performed using Evo M-MLV RT Premix for qPCR (Accurate Biology, AG11706, Changsha, China), and quantitative PCR was conducted using SYBR Green Premix Pro Taq HS qPCR Kit III (Low Rox Plus) (Accurate Biology, AG11739, Changsha, China). Primer sequences are listed in Tables S1 and S2. Relative expression levels were normalized to GAPDH and calculated using the 2^−ΔΔCt^ method.

### 4.7. Preparation of Single-Cell Suspensions and Flow Cytometry Analysis

Bone marrow cells were flushed from bilateral femurs and tibiae with cold PBS, filtered through 70 μm mesh after erythrocyte lysis, and resuspended for counting. Colonic tissues were processed to obtain single-cell suspensions according to a previous study [70]. After removal of adipose tissue and Peyer’s patches, colonic tissues were washed twice with HBSS containing 5 mM EDTA and 1 mM DTT to remove epithelial cells, then digested with 2 mg/mL collagenase type III (Worthington, LS004183, Lakewood, NJ, USA) and 50 μg/mL DNase I (Roche, 10104159001, Basel, Switzerland) in RPMI-1640. Digested samples were filtered through 70 μm cell strainers, enriched by 40% Percoll density gradient centrifugation after erythrocyte lysis, and resuspended in staining buffer. Cell surface staining was performed at 4 °C in the dark following Fc receptor blockade with anti-CD16/32 antibody before cell surface was stained with fluorescent conjugated antibodies. Then, cells were stained with APC anti-mouse CD_3_, AF700 anti-mouse CD_4_, PE anti-mouse TNF-α, PE-Cy7 anti-mouse IL-17A. All antibodies were purchased from Biolegend, San Diego, CA, USA. Cells were analyzed by flow cytometry. Th_17_ cells were defined as CD_3_^+^CD_4_^+^IL-17A^+^ cells. TNF-α^+^ Th_17_ cells were defined as TNF-α-positive cells within the CD_3_^+^CD_4_^+^IL-17A^+^ population (CD_3_^+^CD_4_^+^IL-17A^+^TNF-α^+^).

### 4.8. Metabolite Extraction and UHPLC-Q-TOF MS Analysis

Approximately 50 ± 5 mg of DGME, dried ginger aqueous extract (DGWE), and milk powder control (CON) were homogenized in 400 μL ice-cold methanol/water (4:1, *v*/*v*) containing L-2-chlorophenylalanine (0.02 mg/mL) using a cryogenic grinder (−10 °C, 50 Hz, 6 min), followed by ultrasonic extraction (5 °C, 40 kHz, 30 min). After protein precipitation (−20 °C, 30 min) and centrifugation (13,000× *g*, 15 min, 4 °C), supernatants were collected for LC-MS analysis. Quality control (QC) samples were prepared by pooling 20 μL aliquots from each supernatant.

Metabolite profiling was conducted on a SCIEX TripleTOF 6600 system operating in both positive ion (POS) and negative ion (NEG) modes. Raw data were processed using Progenesis QI (Waters Corporation, Milford, MA, USA) for peak detection, alignment, and annotation against HMDB, Metlin, and in-house libraries. Quantitative data were processed in Progenesis QI for peak detection, retention time alignment, and feature extraction. Data preprocessing included missing value imputation, normalization, and QC-based filtering (features with relative standard deviation > 30% were excluded). The total ion chromatograms (TICs) of the three sample groups in both POS and NEG modes are shown in Figure 4A and Figure S2, and the identification results of the chemical components are summarized in Table 1.

### 4.9. Network Pharmacology Predicts Potential Targets for DGME Treatment of IBD-BL

We applied a network pharmacology strategy to investigate the therapeutic mechanisms of DGME in IBD-BL. First, chemical structures of differential components were collected from PubMed and converted into canonical SMILES representations to ensure structural consistency. All compounds were unified using consistent molecular identifiers for subsequent analyses. Next, putative targets were predicted using SwissTargetPrediction (http://www.swisstargetprediction.ch/, accessed 2 November 2025) based on 2D and 3D similarity. Predicted targets were mapped to official human gene symbols. The targets were then merged and deduplicated to generate a non-redundant compound–target dataset. Based on this dataset, target sets were constructed for each of the 19 compounds. Pairwise Jaccard similarity coefficients were calculated in R (v4.4.2) using dplyr (v1.1.4) and tidyr (v1.3.1) to quantify overlap between target profiles. The similarity matrix was visualized using pheatmap (v1.0.12). This analysis reflects pharmacological similarity and suggests potential functional convergence among compounds. Meanwhile, disease-associated targets for IBD (*n* = 4374) and osteoporosis (*n* = 7707) were collected from GeneCards and DisGeNET. All targets were standardized and deduplicated to ensure dataset consistency. The two disease datasets were intersected to obtain shared targets. These targets were subsequently overlapped with compound targets to define core drug–disease targets. This step represents a key molecular link between DGME and disease mechanisms. Subsequently, core targets were imported into STRING to construct a protein–protein interaction network with a confidence score ≥ 0.7. The network comprised 247 nodes and 1757 edges. The average node degree was 14.23, indicating a highly interconnected structure. Topological analysis was conducted in R using igraph (v2.1.3). Degree centrality was calculated to identify hub targets as major regulatory elements. Finally, Gene Ontology and KEGG enrichment analyses were performed using clusterProfiler (v4.14.4) with gene annotation based on org.Hs.eg.db (v3.20.0). Significantly enriched biological processes and pathways were identified after multiple testing correction.

### 4.10. Western Blot Analysis

Total protein was extracted from colon, bone tissues or cultured cells using RIPA buffer supplemented with protease and phosphatase inhibitors. For phosphorylated proteins, cells were stimulated with LPS (0.5 μg/mL) for 30 min; for cytokine analysis, stimulation lasted 24 h. Lysates were centrifuged at 12,000× *g* for 15 min at 4 °C, and protein concentration was determined by BCA assay (Thermo, 23225, Waltham, MA, USA). Equal amounts of protein (20 μg) were separated by 10% SDS-PAGE and transferred to PVDF membranes. After blocking with 5% non-fat milk, membranes were incubated overnight at 4 °C with primary antibodies against p-MEK, MEK, p-ERK, ERK, and GAPDH (1:1000), followed by HRP-conjugated secondary antibodies (1:5000) for 1 h at room temperature. Protein bands were visualized by enhanced chemiluminescence (ECL) and quantified using ImageJ software.

### 4.11. DNA Extraction, 16S rRNA Gene Sequencing, and Data Analysis

Total genomic DNA was extracted from the feces of all samples using the QIAamp^®^ Fast DNA Stool Mini Kit (51604; Qiagen, Hilden, Germany) following the manufacturer’s protocol. The V3–V4 region of the bacterial 16S rRNA gene was amplified using universal primers 338F (ACTCCTACGGGAGGCAGCAG) and 806R (GGACTACHVGGGTWTCTAAT). PCR products were purified and sequenced on an Illumina NovaSeq 6000 platform (paired-end, 2 × 250 bp). Raw sequences were demultiplexed, quality filtered, and merged using FLASH, Cutadapt, and fastp. Chimeric sequences were removed using UCHIME against the SILVA database. High-quality reads were processed in QIIME2 (v2021.4) with the DADA2 plugin to generate amplicon sequence variants (ASVs) and a corresponding feature table. Taxonomic classification was performed using the SILVA 138.1 database.

Alpha diversity indices were calculated to evaluate microbial richness and diversity. The Chao1 index was used to estimate species richness based on rare taxa. The Shannon index was used to reflect overall diversity by considering both richness and evenness. Pielou_e was used to measure community evenness. Beta diversity was calculated using Bray–Curtis distance, which accounts for abundance differences, and Jaccard distance, which is based on presence–absence information. PCoA was performed to visualize differences in community structure among groups. Differential taxa were identified using LEfSe (LDA threshold > 2), combining statistical significance with biological relevance. Community variation among groups was further evaluated using ANOSIM and PERMANOVA based on distance matrices to assess the significance of group separation.

### 4.12. Fecal Metabolite Extraction, Untargeted Metabolomics Detection, and Data Processing

Fecal samples were collected from Control, DSS, and DGME groups (*n* = 6 per group) at sacrifice and stored at −80 °C. Approximately 100 mg of each sample was extracted with 500 µL of pre-chilled 80% methanol. Samples were vortexed, incubated on ice for 5 min, and centrifuged at 15,000× *g* for 20 min at 4 °C. The supernatant was diluted with LC-MS-grade water to a final methanol content of 53% and centrifuged again. The resulting supernatant was filtered through a 0.22 µm membrane prior to analysis. Metabolomic profiling was performed using a Vanquish UHPLC system coupled with a Q Exactive HF-X Orbitrap mass spectrometer. Chromatographic separation was achieved on a Hypersil Gold C18 column (100 × 2.1 mm, 1.9 µm) at 40 °C with a methanol–water gradient containing 0.1% formic acid at a flow rate of 0.2 mL/min. Data were acquired in both positive and negative ion modes using data-dependent MS/MS scanning over an *m*/*z* range of 100–1500.

Raw data were converted to mzXML format using ProteoWizard and processed using XCMS for peak detection, retention time alignment, and feature extraction with a mass tolerance of 10 ppm. This step generated a data matrix consisting of retention time, *m*/*z*, and peak intensity. Metabolites were annotated by matching MS/MS spectra against HMDB, KEGG, and LipidMaps databases to improve identification confidence. To ensure data quality, background ions were removed using blank samples. Peak intensities were normalized based on QC samples to correct for signal drift and batch effects. Features with a coefficient of variation > 30% across QC samples were excluded to improve data reliability and reproducibility. Multivariate statistical analyses were performed using metaX. Multivariate analyses including PLS-DA were conducted, and differential metabolites were identified based on *p* < 0.05, VIP > 1.0 and fold change > 1.2 or <0.833 followed by KEGG pathway enrichment analysis. Spearman correlation coefficients between microbes and metabolites were inferred using the “psych” package in R.

### 4.13. Effects of Vanillin and Spermidine on LPS-Induced Acute Injury in Jurkat T Cells

The human acute T-cell leukemia cell line (Jurkat) was obtained from the Cell Resource Center of Shanghai for Biological Science, Chinese Academy of Sciences (Shanghai, China). Cells were cultured in RPMI 1640 complete medium supplemented with 10% fetal bovine serum (FBS), and 1% penicillin/streptomycin in a 37 °C, 5% CO_2_ incubator. The cells were passaged every 2–3 days, and cells in the logarithmic growth phase were used for subsequent experiments.

Cell viability was measured by CCK-8 assay. Jurkat T cells in the logarithmic growth phase were seeded into 96-well plates at a density of 5 × 10^4^ cells per well and incubated at 37 °C for 24 h. Cells were then treated with vanillin (VAN) at concentrations of 25, 50, 100, 200, 400, and 800 μM or spermidine (SPD) at 0.1, 1, 10, 50, 100, and 1000 μM, respectively. After 24 h incubation, 10 μL of CCK-8 reagent was added to each well, and the cells were further incubated at 37 °C for 2–4 h. The optical density (OD) at 450 nm was measured using a microplate reader, and cell viability was calculated based on the OD values.

Cells were divided into five groups: Control, LPS (0.5 μg/mL), VAN (100 μM), SPD (10 μM), VAN + SPD (50 μM + 5 μM). Jurkat T cells were seeded in 6-well plates at a density of 5 × 10^6^ cells/well. Except for the Control group, cells were pretreated with the respective drugs for 24 h prior to LPS stimulation.

### 4.14. Statistical Analysis

Statistical analyses of validation experiments were performed using GraphPad Prism (version 9.0.0), while computational and transcriptomic analyses were conducted in R (version 4.2.2). Parametric data were analyzed using an unpaired Student’s *t*-test or one-way analysis of variance (ANOVA) with Tukey’s post hoc test. Non-parametric data were analyzed using the Mann–Whitney U-tests or Kruskal–Wallis test. A *p* value of less than 0.05 was considered statistically significant. Data were presented as median or mean ± standard error of the mean (mean ± SEM).

## 5. Conclusions

DGME effectively ameliorates IBD-BL through integrated modulation of the gut–bone axis. It restructures gut microbiota composition by enriching beneficial genera (*Lactobacillus*, *Anaerotruncus*, *Massiliomicrobiota*) and depleting pro-inflammatory taxa (*Romboutsia*, *Turicibacter*). This microbial remodeling increases production of VAN and SPD. These metabolites coordinately suppress MEK/ERK phosphorylation in pathogenic TNF-α^+^ Th17 cells. Consequently, osteoclast-mediated bone resorption is attenuated. This study provides novel mechanistic insights into how milk-extracted botanicals modulate osteoimmunology through sensing of microbiota-derived metabolites. VAN and SPD emerge as functional mediators connecting dietary ginger to bone protection. However, the current evidence remains associative. Causal roles of specific taxa and metabolites require validation through fecal microbiota transplantation in gnotobiotic models. Clinical translation also necessitates human cohort validation and dosage optimization.

## Figures and Tables

**Table 1:** UHPLC-Q-TOF MS identified the main differential compounds between DGME and DGWE.

No.	Identified Compounds	M/Z	RT/min	Mass Error	Adducts	Formula
1	6-hydroxyshogaol	293.1723	12.77	−8.25	M + H	C_17_H_24_O_4_
2	10-shogaol	333.24	13.51	−7.37	M + H	C_21_H_32_O_3_
3	[8]-dehydroshogaol	320.2196	12.55	−7.97	M + NH_4_	C_19_H_26_O_3_
4	8-shogaol	305.2102	13.2	−3.15	M + H	C_19_H_28_O_3_
5	6-shogaol	275.1652	9.89	−0.17	M − H	C_17_H_24_O_3_
6	demethoxyshogaol	247.1688	9.73	−1.67	M + H	C_16_H_22_O_2_
7	10-gingerol	349.2378	13.04	−1.67	M − H	C_21_H_34_O_4_
8	[12]-gingerol	361.2718	13.57	−5.14	M + H − H_2_O	C_23_H_38_O_4_
9	Gingerol	293.1758	9.7	0.03	M − H	C_17_H_26_O_4_
10	6-gingerol	293.1757	9.89	−0.47	M − H	C_17_H_26_O_4_
11	[8]-paradol	307.2243	12.14	−8.2	M + H	C_19_H_30_O_3_
12	[7]-paradol	293.2092	12.4	−6.52	M + H	C_18_H_28_O_3_
13	[8]-dehydrogingerdione	319.1881	12.55	−7.26	M + H	C_19_H_26_O_4_
14	[10]-dehydrogingerdione	347.2203	13.8	−3.99	M + H	C_21_H_30_O_4_
15	1-dehydro-[6]-gingerdione	289.1443	12.86	−0.7	M − H	C_17_H_22_O_4_
16	[6]-dehydrogingerdione	291.1588	12.9	−1.15	M + H	C_17_H_22_O_4_
17	[12]-gingerdione	375.2539	13.26	−0.54	M − H	C_23_H_36_O_4_
18	[10]-gingerdione	349.2371	14.15	−0.75	M + H	C_21_H_32_O_4_
19	[6]-gingerdione	291.1597	8.47	−1.5	M − H	C_17_H_24_O_4_

## Data Availability

The original contributions presented in this study are included in the article/Supplementary Materials. Further inquiries can be directed to the corresponding author.

## Supplementary Materials

The following supporting information can be downloaded at: https://www.mdpi.com/article/10.3390/ph19050675/s1, Figure S1: DGME suppresses Th17 cell populations in colon and bone marrow. Figure S2: UHPLC-Q-TOF MS-based comparative metabolomic profiling of DGME and DGWE. Figure S3: Effects of DGME and DGWE on bone loss in DSS-induced IBD-BL mice. Figure S4: Network-based identification of MEK/ERK signaling as a key pathway of DGME. Figure S5: DGME remodels gut microbiota–metabolite of IBD-BL mice. Figure S6: Effects of vanillin and spermidine on Jurkat T cell viability; Table S1: RT-qPCR primer sequences in animal experiments. Table S2: RT-qPCR primer sequences in Jurkat T cell experiments. Table S3: Cytotoxicity assessment of vanillin in Jurkat T cells. Table S4: Cytotoxicity assessment of spermidine in Jurkat T cells (*n* = 3).

## Abbreviations

The following abbreviations are used in this manuscript:

ADW	Autoclaved drinking water
BS/BV	Bone surface to bone volume ratio
BS/TV	Bone surface density
BV/TV	Bone volume fraction
CD	Crohn’s disease
DAI	Disease activity index
DGME	Dried ginger milk extract
DGWE	Dried ginger aqueous extract
DSS	Dextran sulfate sodium
ERK	Extracellular signal-regulated kinase
FC	Fold change
FMH	Food–medicine homology
GMP	Guanosine monophosphate
GO	Gene Ontology
H&E	Hematoxylin and eosin
IBD	Inflammatory bowel disease
IBD-BL	Inflammatory bowel disease-associated bone loss
KEGG	Kyoto Encyclopedia of Genes and Genomes
LDA	Linear discriminant analysis
LPS	Lipopolysaccharide
MAPK	Mitogen-activated protein kinase
MEK	Mitogen-activated protein kinase kinase
M-CSF	Macrophage colony-stimulating factor
NFATc1	Nuclear factor of activated T cells 1
OC.N/BPm	Osteoclast number per bone perimeter
OC.S/BS	Osteoclast surface per bone surface
PCoA	Principal coordinate analysis
PLS-DA	Partial least squares discriminant analysis
RANKL	Receptor activator of nuclear factor kappa-B ligand
SASP	Sulfasalazine
SMI	Structural model index
SPD	Spermidine
Tb.N	Trabecular number
Tb.Sp	Trabecular separation
Tb.Th	Trabecular thickness
TCMSP	Traditional Chinese Medicine Systems Pharmacology database
Th17	T helper 17 cell
TIC	Total ion chromatogram
TNF-α	Tumor necrosis factor-alpha
UC	Ulcerative colitis
UHPLC-Q-TOF MS	Ultra-high-performance liquid chromatography coupled with quadrupole time-of-flight mass spectrometry
VAN	Vanillin
VIP	Variable importance in projection
