# Supplementary material for: Dried Ginger Milk Extract Alleviates Inflammatory Bowel Disease-Associated Bone Loss via Gut Microbiota–Metabolite Remodeling and MEK/ERK Inhibition
Source: Pharmaceuticals (Basel). 2026 Apr 26;19(5):675. doi: 10.3390/ph19050675 (PMC13209199; doi:10.3390/ph19050675)
Supplement: Supplementary file 1 [file pharmaceuticals-19-00675-s001.zip › pharmaceuticals-4256603-supplementary.pdf]

Dried Ginger Milk Extract Alleviates Inflammatory Bowel Disease-Associated Bone Loss via Gut Microbiota–Metabolite Remodeling and MEK/ERK Inhibition

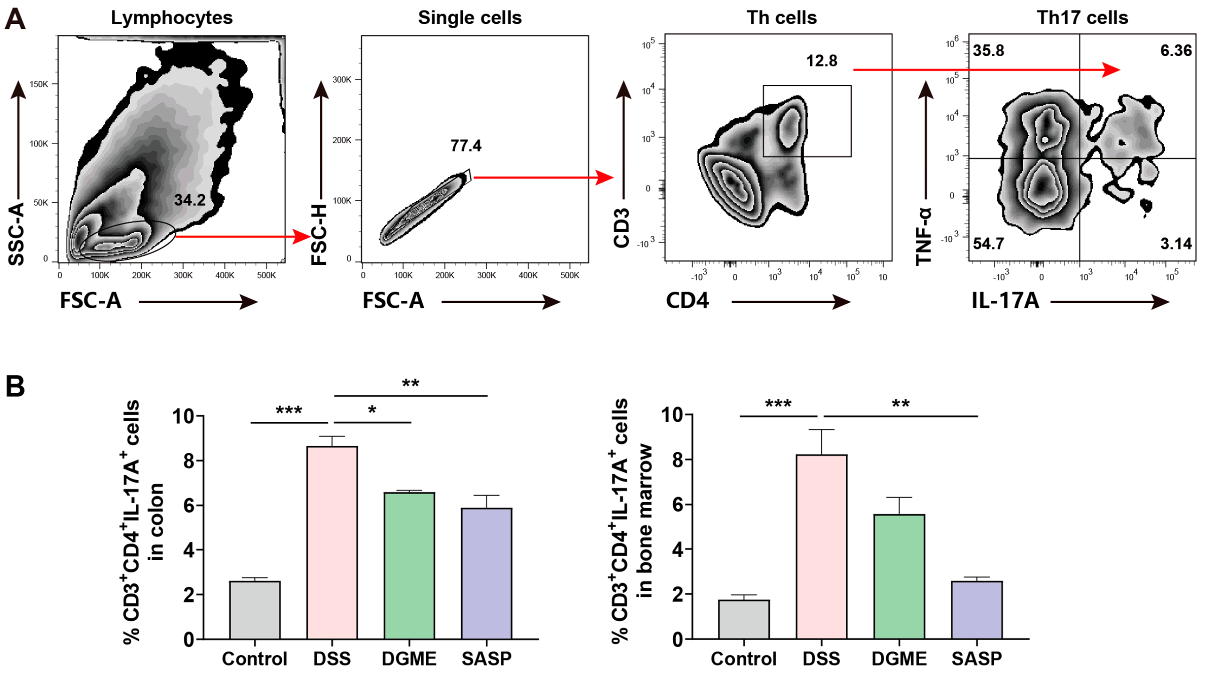

Figure S1. DGME suppresses Th17 cell populations in colon and bone marrow. (A) Gating strategy for the analysis of $CD4^{+}$ T cell subsets. (B) The proportions of Th17 cells ($CD3^{+}CD4^{+}IL\text{-}17A^{+}$) in colon and bone marrow. Data are presented as mean ± SEM. $^{*}p < 0.05$, $^{**}p < 0.01$, $^{***}p < 0.001$.

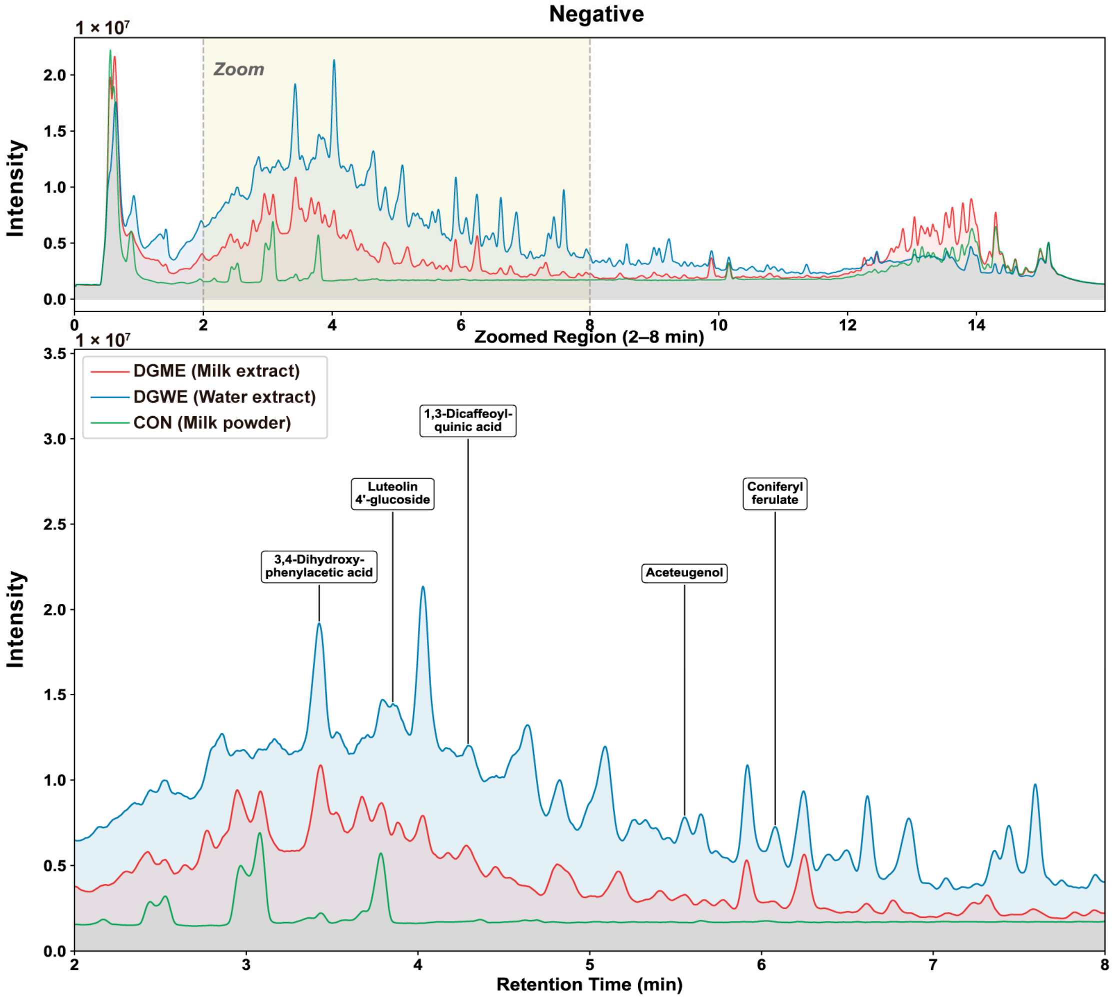

Figure S2. UHPLC-Q-TOF MS–based comparative metabolomic profiling of DGME and DGWE. Total ion chromatograms of the three samples in negative ion mode.

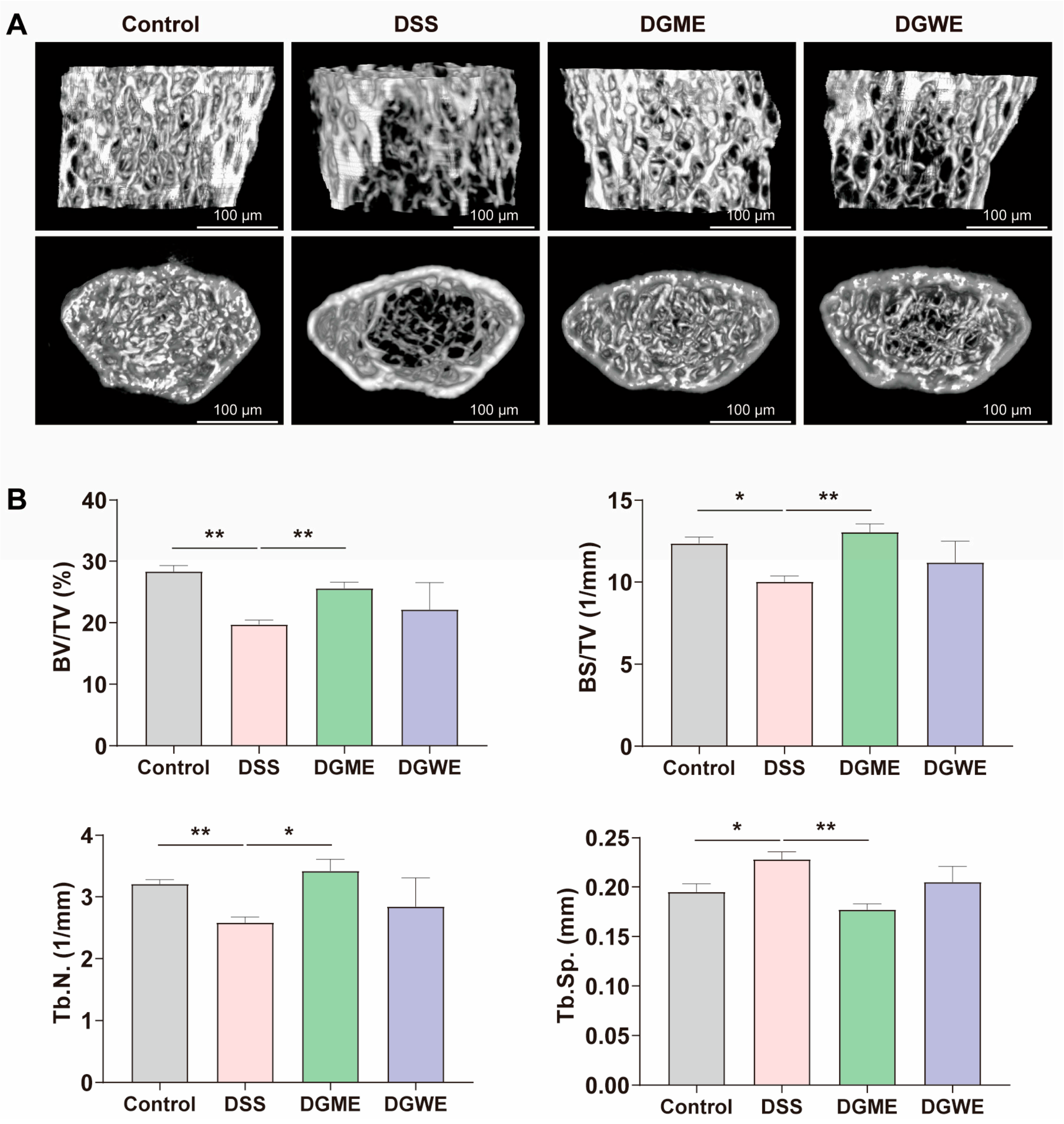

Figure S3. Effects of DGME and DGWE on bone loss in DSS-induced IBD-BL mice. (A) Representative micro-CT three-dimensional reconstruction of distal femoral trabecular bone. (B) Quantitative micro-CT analysis of trabecular bone parameters, including bone volume fraction (BV/TV), bone surface density (BS/TV), trabecular number (Tb.N.), and trabecular separation (Tb.Sp.). Data are presented as mean ± SEM. Statistical significance was determined by comparison with the DSS group. **p* < 0.05, ***p* < 0.01.

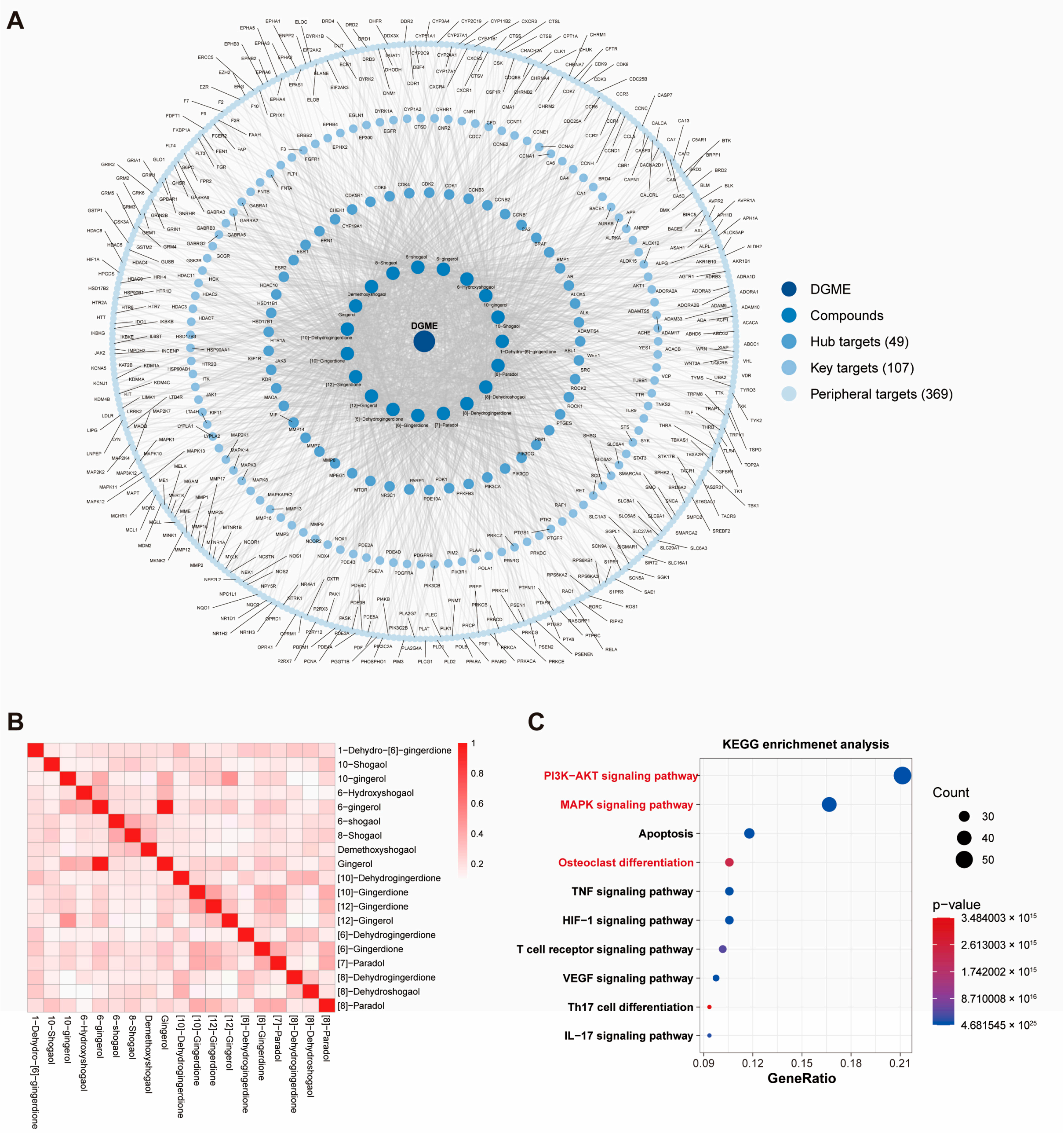

Figure S4. Network-based identification of MEK/ERK signaling as a key pathway of DGME. (A) Compound–target network of 19 bioactive components. (B) Jaccard similarity analysis of target overlap. (C) KEGG enrichment analysis.

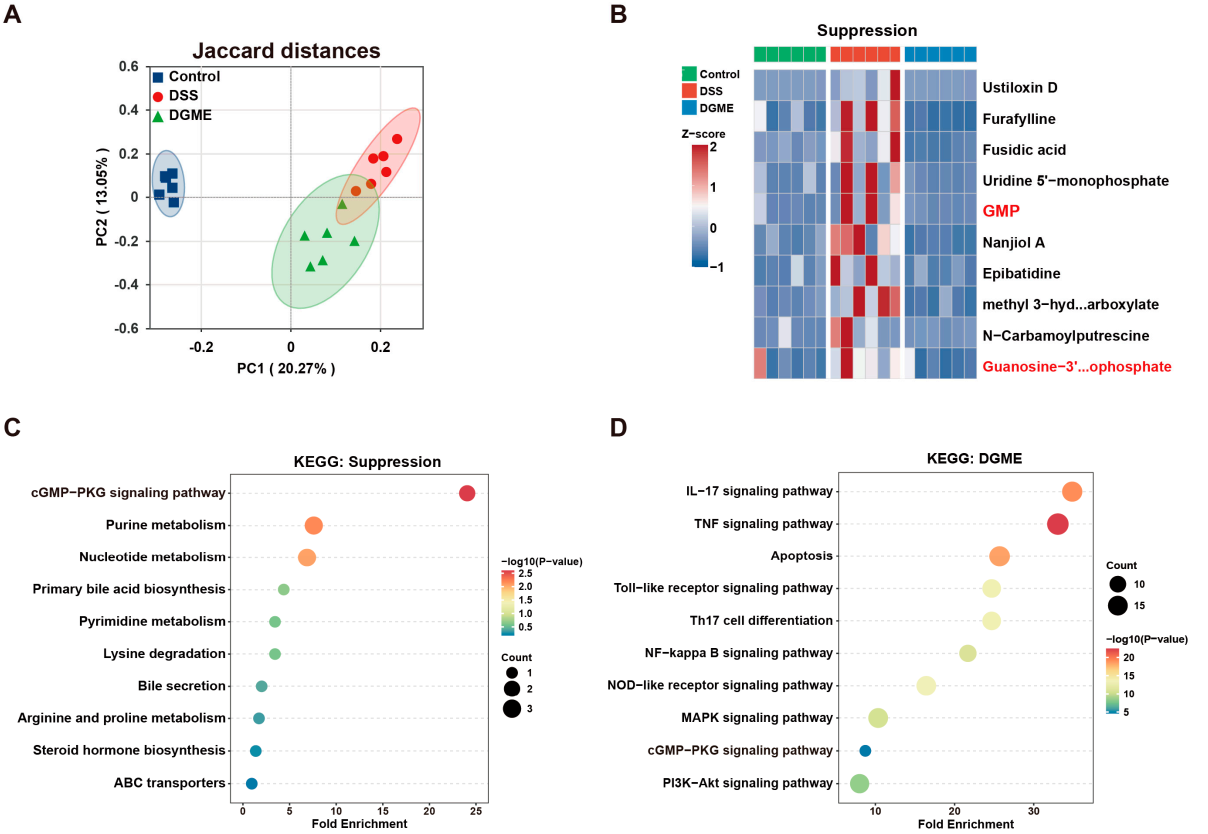

Figure S5. DGME remodels gut microbiota–metabolite of IBD-BL mice. (A) Beta-diversities of gut microbiota profiles illustrated with PCoA using Jaccard distances. (B) Z-score heatmap of suppression-pattern metabolites. (C) KEGG enrichment analysis of suppression metabolites. (D) KEGG pathway enrichment of DGME-predicted targets. Differential metabolites were screened using $p < 0.05$, VIP > 1.0 and fold change > 1.2 or < 0.833.

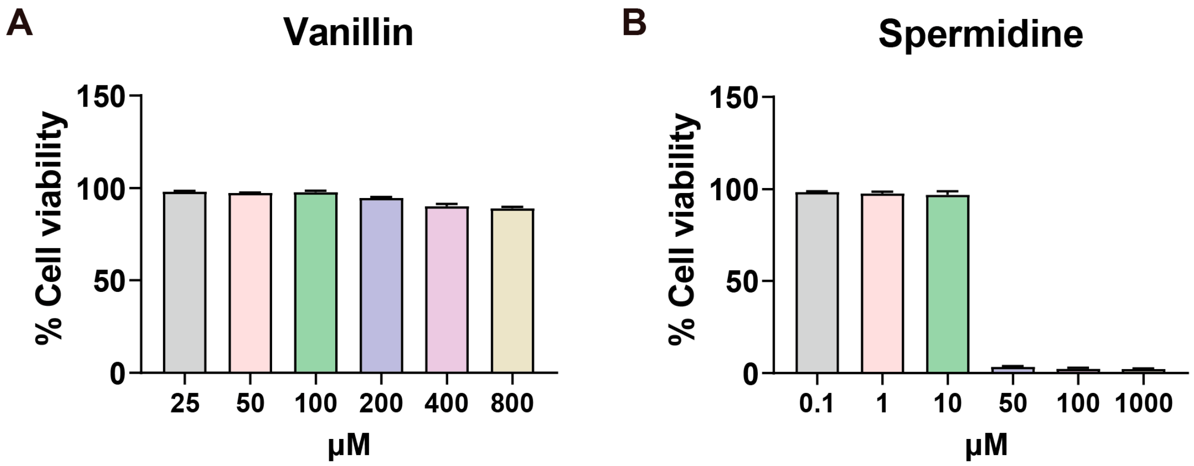

Figure S6. Effects of vanillin and spermidine on Jurkat T cell viability. (A–B) Cytotoxicity of various concentrations of vanillin (A) and spermidine (B) against Jurkat T cells was assessed using the CCK-8 assay to determine the optimal dosage.
